# Synthesis of Fe-TiO_2_ and Cu-TiO_2_ Based Materials by Olive Leaves Biotemplating—Application to Hydrogen Production from Glycerol Photoreforming

**DOI:** 10.3390/nano13040664

**Published:** 2023-02-08

**Authors:** Juan Martín-Gómez, Susana Reca-Expósito, Francisco J. López-Tenllado, Jesús Hidalgo-Carrillo, Alberto Marinas, Francisco J. Urbano

**Affiliations:** Departamento de Química Orgánica, Instituto Químico para la Energía y el Medioambiente (IQUEMA), Universidad de Córdoba, E-14071 Córdoba, Spain

**Keywords:** hydrogen production, biotemplating, olive leaf, titanium dioxide, copper, iron

## Abstract

Hydrogen production is mainly based on the use of fossil fuels, but currently, many alternative routes are being developed, among which the photo-reforming of oxygenated organic compounds stands out. Recently, several studies have been carried out in order to develop new techniques to create bio-inspired TiO_2_ structures. One of these is ‘biotemplating’, a process that replicates a biological system in an inorganic TiO_2_-based structure. In this study, olive by-products—olive leaves—are valorized as a biotemplate for the synthesis of new Fe-TiO_2-_ and Cu-TiO_2_-based photocatalysts with the aim of improving the replication of the leaf structure and enhancing hydrogen photoproduction. In conclusion, the incorporation of iron and copper decreases the band gap and increases the energetic disorder at the band edges. Moreover, it is verified by SEM and TEM that the metals are not found forming particles but are introduced into the formed TiO_2_ structure. The accuracy of the internal and external structure replication is improved with the incorporation of Fe in the synthesis, while the incorporation of Cu substantially improves the production of hydrogen, which is multiplied 14 times under UV light and 6 times under sunlight, as compared to a pure TiO_2_ structure.

## 1. Introduction

In photocatalysis, TiO_2_ is one of the most widely used and studied catalysts due to its properties, such as low cost, abundance, photochemical stability and low toxicity, etc. [1,2]. However, it has two major drawbacks: a fast recombination of electron-hole pairs and its limited absorption capacity in the visible region. These limitations have been studied in numerous studies, using different techniques to minimize them such as metal doping, non-metal doping, co-doping and coupling with other semiconductors or surface modification through organic materials, among others [3,4,5,6,7,8,9,10,11].

In contrast, materials with a multitude of different structures and inspiring properties for the creation of new catalysts, such as sophistication, miniaturization, hierarchical organization, hybridization, strength and adaptability, can be found in nature [12]. The technique used for the replication of structures or the use of templates from nature is called ‘biotemplating’. This technique makes it possible to obtain materials whose properties and complexity can be controlled at the structural level. Thus, biotemplating can be used to create different TiO_2_-based structures to improve their activity and mitigate their limitations. Some examples of TiO_2_-based catalysts that replicate structures from nature can be found in the literature. Mohamed et al. developed mesoporous TiO_2_ nanotubes replicating the structure of cellulose, thus obtaining a catalyst with high catalytic efficiency for organic pollutant removal from wastewater upon illumination with visible light [13]. Xiao et al. synthesized cerium-doped mesoporous TiO_2_ nanofibers by replicating the structure of collagen fibers. With this structure, they increased the visible light absorption capacity and improved the catalyst activity in the degradation of Rhodamine B under visible light [14]. Bu et al. created hollow spheres formed by copper and TiO_2_, using rapeseed pollen as a biotemplate. In this study, they managed to increase the visible light absorption of the catalysts, increasing their activity in the degradation of chlortetracycline hydrochloride under visible and UV lights [15]. Li et al. created microtubes formed of intertwined nanofibers based on tin-doped TiO_2_ using cellulosic cotton as a biotemplate, achieving enhanced visible light absorption by TiO_2_ that was tested in the degradation of methylene blue [16]. Therefore, the use of biological templates that confer a defined structure, together with the use of metals, allows the photocatalytic properties of TiO_2_ to be improved.

Currently, the European Union is the largest producer of olive oil, accounting for 68% of world production. During this production, almost 10 million tons of by-products such as olive pomace or olive leaves are generated in the industrial sector and approximately another three million in the agricultural sector, of which 25% are leaves [17]. In Spain, Andalusia is the autonomous community with the most hectares of olive production and cultivation, approximately 1,500,000 hectares, which are continuously increasing due to the development in agriculture and irrigation techniques [18]. Olive leaves, derived from the industrial sector or from pruning processes, represent a high number of by-products at national level that could be used in other processes, giving them a second useful life. The olive tree Olea europea L. is a tree of the Oleaceae family cultivated in the temperate zones of the planet, where 95% of the world’s cultivated area is in the Mediterranean zone. It is a C3 plant that fixes CO_2_ directly in the Calvin cycle. The leaves are lanceolate in shape, and at a structural level different cell layers can be differentiated. In the beam zone, the first layer of cells that can be distinguished is the epidermis, formed by cells that act as lenses and concentrate the light. As we move towards the underside of the leaf, we find the palisade parenchyma, where most of the chloroplasts are concentrated. This organelle is mainly responsible for the uptake of blue and red light through the action of chlorophyll. In addition, in the palisade parenchyma, the phenomena known as the sieve effect and light channeling take place. These phenomena are responsible for transmitting light to the other parts of the leaf. Finally, we find the lagoon parenchyma, containing many air spaces, where gas exchange takes place and light absorption is increased due to the phenomena of light reflection and refraction [12].

These characteristics of olive leaves are very attractive from a photocatalytic point of view since they allow focusing and scattering light inside the catalyst. In two previous studies, the bio-imitation of olive leaves with TiO_2_ and with TiO_2_ and CuO was studied, where it could be verified that the replication of the olive leaf structure allows the production of hydrogen from glycerol to be improved with respect to a commercial TiO_2_, and that CuO forms a heterojunction with TiO_2_, greatly improving the activity [19,20]. However, no metal prioritizing the enhancement of olive leaf replication has been studied. Iron in this aspect is a metal that is used in photocatalysis as a cocatalyst and presents a predisposition to be introduced into the porphyrin group of chlorophyll replacing magnesium, since iron is naturally part of the porphyrin group of hemoglobin. Therefore, the objective of this study is the design and synthesis of new photocatalysts valorizing olive leaves, a by-product of olive growing. For this purpose, the internal and external structure of the leaf is replicated based firstly on TiO_2_ and adding as co-catalysts copper, to improve the activity under UV and visible light, and iron, which, using structural memory, will allow for a more accurate replication of the leaf structure, thus improving the diffusion of light by the catalyst.

## 2. Materials and Methods

### 2.1. Catalyst Synthesis

**Figure 1:**
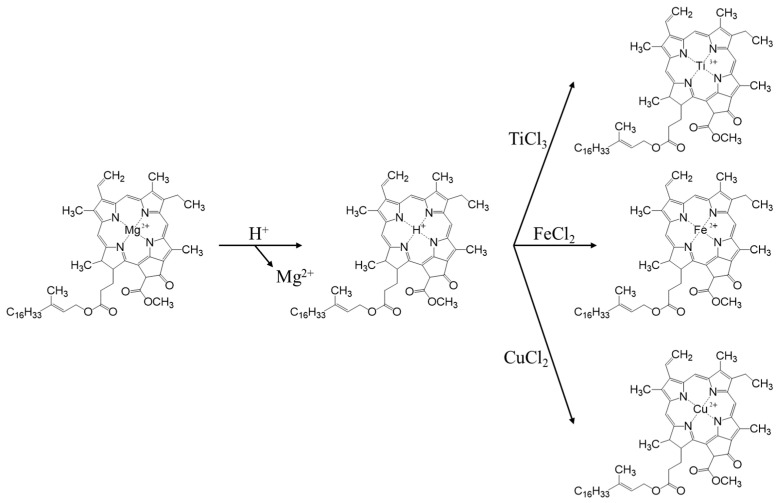
Pheophorbide synthesis route.

The replication of the nano- and microstructures of olive leaf photosystems was carried out using ion exchange to mimic the natural process of chlorophyll degradation, according to a synthetic procedure optimized in a previous study [19]. The process is composed of three phases, the first of which consists of an acid treatment to eliminate metal cations, mainly the Mg^2+^ of the porphyrins, which will be replaced by H^+^, forming yellow-brown pheophytins (Figure 1). For this first step, fresh olive leaves are washed with MiliQ water and dried. From these leaves, the central rib is removed along with the petiole and the tip, and they are cut into fragments which are as small as possible. Subsequently, 10 g of chopped leaves are taken, placed in a round bottom flask along with 150 mL of 5% HCl and left in agitation overnight under inert atmosphere.

In a second step, 30 mL of TiCl_3_ (0.061 mol) is injected through the septum. In this process, ion exchange takes place, where protons from the pheophytin group of the chlorophyll are exchanged for Ti^3+^, which is thus introduced into the nanostructure of the thylakoids (Figure 1). In the third phase, the Ti^3+^ ions act as seeds for the subsequent formation of a TiO_2_ structure, which replicates the structure of the olive leaf. For this purpose, the leaves are filtered with a Büchner funnel, washed 3 times with 50 mL of Milli-Q water and dried in the vacuum desiccator at 80 °C. When the leaves are completely dry, they are returned to the flask and placed in suspension with 98 mL of isopropanol and shaken overnight to remove any remaining water. Then, they are again filtered, washing on this occasion with isopropanol, and are reintroduced to a flask with 98 mL of isopropanol and 9 mL of titanium isopropoxide and shaken overnight. After this agitation time, it was subjected to reflux at 95 °C for 6 h and finally, the dry solid obtained from the synthesis was calcined in a muffle at 550 °C for 6 h with a temperature ramp of 1 °C/min. The nomenclature used for this catalyst was AOL, from the acronym ‘Artificial Olive Leaf’.

In order to evaluate the effect of the incorporation of metals, the synthesis of two new catalysts was developed by introducing iron and copper to the olive leaf. These syntheses follow the same guidelines as the one previously developed, only with the particularity that instead of adding 30 mL of TiCl_3_, 30 mL of FeCl_2_ or CuCl_2_ were added, under the same concentration conditions (0.061 mol). These catalysts are called Fe-AOL and Cu-AOL, respectively.

### 2.2. Characterization of the Catalyst

To determine the morphology, structure and composition of the different catalysts synthesized by the biotemplating process, several characterization techniques were carried out.

#### 2.2.1. X-ray Diffraction (XRD)

X-ray diffraction was carried out on a Bruker D8 Discover (Bruker Española S.A., Madrid, Spain) with monochromatic CuKα1 source at a radiation λ = 1.54 Å in an angular range of 10–80° and with a scan rate of 1.45° 2Ɵ-min^−1^.

#### 2.2.2. Ultraviolet-Visible Spectroscopy (UV-Vis)

Diffuse reflectance spectra were obtained using an Agilent Cary 5000 UV-Vis-NIR spectrophotometer (Agilent, Santa Clara, CA, USA). The forbidden band values were obtained from the plot of the Kubelka-Munk function [F(R) × E]^1/2^ versus the energy of the absorbed light.

#### 2.2.3. X-ray Photoelectron Spectroscopy (XPS)

X-ray photoelectron spectroscopy was carried out on a Leibold-Heraeus LHS10 spectrometer (SPECS, Berlin, Germany), which had a chamber capable of operating at less than 2 × 10^−9^ Torr, equipped with an EA-200MCD hemispherical electron analyzer with an AlKα X-ray source (hν = 1486.6 eV) at 120 W, 30 mA, with C (1 s) as reference energy (284.6 eV). The equipment is available at the Central Service for Research Support (SCAI) of the University of Cordoba.

#### 2.2.4. Scanning Electron Microscopy (SEM)

Scanning electron micrographs were obtained using a JEOL JSM 7800F Prime microscope (JEOL Ltd., Tokyo, Japan). The equipment is available at the Central Service for Research Support (SCAI) of the University of Córdoba.

#### 2.2.5. X-ray Fluorescence (XRF)

X-ray fluorescence was carried out on a Rigaku ZSK Priums IV wavelength X-ray spectrometer (Rigaku, Austin, TX, USA). It is equipped with a 3 kW Rh-target X-ray tube, ten analyzer crystals, a proportional gas counter for light element detention and a scintillation counter for heavy elements. 

#### 2.2.6. Glycerol Photo-Reforming Reaction

**Figure 2:**
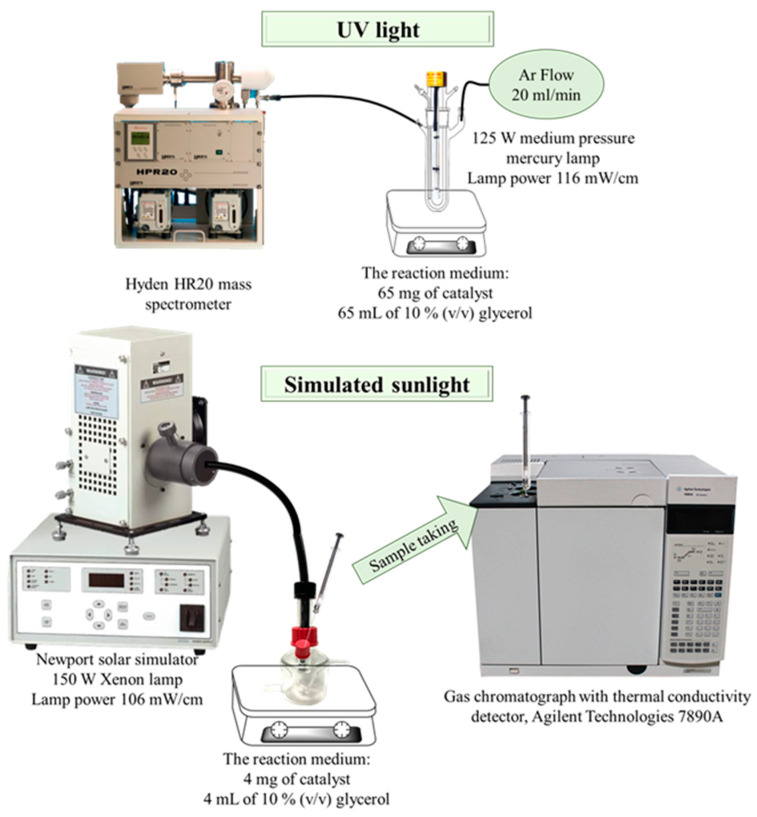
Scheme of experimental set-up.

The synthesized catalysts were tested in the hydrogen production reaction by photocatalytic reforming of 10% (*v/v*) glycerol in water, with two experimental designs with different light sources (Figure 2).

The experiment under UV light was carried out in a Pyrex double-walled cylindrical immersion reactor (23 cm long × 5 cm internal diameter, total volume 190 mL) with a 125 W medium pressure mercury lamp (Photochemical Reactors Ltd., Reading, UK). In a typical experiment, 65 mg of catalyst were suspended in 65 mL of 10% (*v/v*) glycerol and the mixture was bubbled with an Ar flow (20 mL/min) along the whole process. The composition of the reactor outlet gases was analyzed on-line on a Hyden HR20 mass spectrometer (Hyden Analytical Ltd., Warrington, UK), where H_2_ and CO_2_ were continuously monitored (m/z values of 2 and 44, respectively). H_2_ and CO_2_ were quantified through the calibration of the mass spectrometer with a certified gas bottle containing 2% H_2_, 1% CO_2_ and 97% Ar.

For the experiments under simulated sunlight, the reactions were carried out in an 8.5 mL cylindrical reactor under Ar atmosphere. The reactor was irradiated with a Newport solar simulator equipped with a 150 W Xenon lamp. The reaction medium consisted of 4 mg of catalyst suspended on 4 mL of 10 % (*v/v*) glycerol, leaving a headspace volume in the reactor of 4.5 mL where gas products accumulated. Samples for analysis were drawn with a precision analytical syringe with a pressure seal (Valco VICI Precision Syringes, 1 mL, leak-tight to 250 psi) from the reactor headspace. Samples were analyzed on a gas chromatograph with thermal conductivity detector, Agilent Technologies 7890A (Agilent, Santa Clara, CA, USA) equipped with a Supelco Carboxen 1010 column (Sigma-Aldrich, Darmstadt, Germany). H_2_ was quantified using a calibration line manually spiked with pure H_2_ in the gas chromatograph, with a concentration range from 0 to 20 µmol.

The power of the lamps in the sample compartments was measured at <800 nm with an Ophir Starlite equipment, being 116 mW/cm and 106 mW/cm for the UV lamp and solar simulator, respectively.

## 3. Results and Discussions

### 3.1. Synthesis of Catalysts and Morphological Analysis by SEM and TEM

The anatomical structure of the leaf is formed, in the upper mesophyll, of two or three layers of long and compact cells that form the palisade parenchyma and, in the lower mesophyll, of the spongy parenchyma, which has a greater part aerated by intercellular spaces [21]. This structure gives leaves the ability to absorb and scatter sunlight. For this reason, olive leaves have been used as a template for the synthesis of new titania-based catalysts referred to as artificial olive leaves. 

In this study, three catalysts were synthesized using a biomimetization procedure that aims to replicate the internal and external structure of the olive leaf with a structure based on titanium (AOL), on titanium and iron (Fe-AOL) and on titanium and copper (Cu-AOL). During the first step of the synthetic process, Mg^2+^ was removed from the porphyrin ring and substituted with a proton. Afterwards, in a second step, the Ti^3+^, Cu^2+^ or Fe^2+^ cations were introduced, leading to the intermediate precursors that were used as seeds for the TiO_2_ structure growing from Ti(iPrO)_4_ hydrolysis. Finally, calcination led to the AOL, Cu-AOL, and Fe-AOL solids.

**Figure 3:**
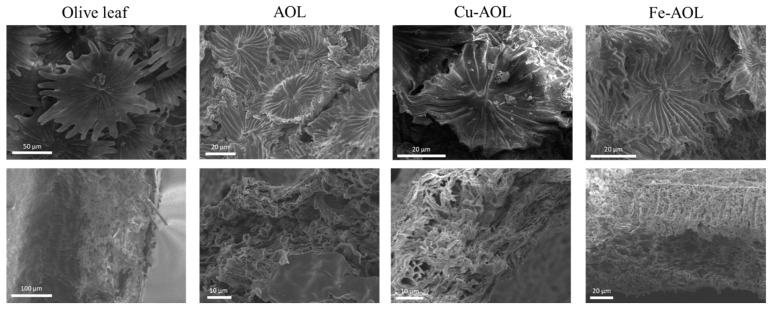
SEM micrographs of trichomes (**upper** images) found on the underside of leaves and a cross section of leaves (**bottom** images) for the olive leaf and the mimetic AOL, Fe-AOL and Cu-AOL solids synthesized.

To analyze the surface morphology of the catalysts and check if the structure of the leaves had been replicated in the solids, scanning electron spectroscopy (SEM) was used, and the results are summarized in Figure 3 and Supplementary Figures S1–S3. In the upper images of Figure 3, the trichomes, a typical structure of the underside of the olive leaf, with a stellate morphology, can be observed in both olive leaves and the synthesized catalysts. However, in the synthesized catalysts, the trichomes present are smaller in size and showed a narrowing in the appendages because of the dehydration of the leaf and the loss of organic matter. Despite this, the trichomes are clearly observed and it was verified that they had been replicated in each catalyst, showing the correct performance of the biotemplated technique. In addition, if a cross section of the solids is analyzed (Figure 3, bottom images), as was the case previously, the thickness of the fresh leaf is reduced in the synthesized catalysts. Nevertheless, the characteristic channeling and sponginess of the olive leaf are perfectly replicated in all the catalysts. Figure 3 also revealed that, among the synthesized solids, the Fe-AOL is the one that best replicates the internal structure of the olive leaf, since it allows both the palisade parenchyma and the spongy parenchyma characteristic of the olive leaf mesophyll to be perfectly identified. This could occur because Fe^2+^ can be found naturally in the porphyrin group of blood hemoglobin, which reflects its suitability as part of this structure and, therefore, a greater ease of exchange to enter the porphyrin ring of chlorophyll can be assumed compared to the other metals (Ti^3+^ and Cu^2+^). On the other hand, Supplementary Figures S1–S3 show the results of the SEM-EDS analyses performed at a higher magnification for the synthesized catalysts. These figures show that both Fe and Cu are homogeneously distributed throughout the TiO_2_ structure that replicates the olive leaf, not observing the formation of agglomerates of Fe or Cu oxides in the form of nanoparticles.

**Figure 4:**
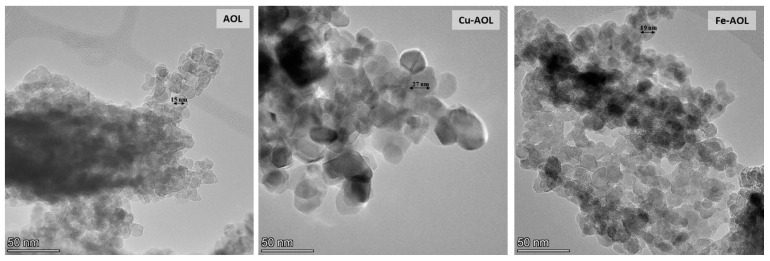
TEM micrographs of the mimetic AOL, Cu-AOL and Fe-AOL solids.

In order to confirm the absence of iron or copper oxide nanoparticles in the Fe-AOL and Cu-AOL catalysts, the solids were also analyzed by TEM and STEM-EDS, obtaining the results shown in Supplementary Figures S4–S7. These figures confirm the absence of these nanoparticles in the catalysts, as the Fe or Cu cations are incorporated into the TiO_2_ structure that constitutes the catalyst matrix. Moreover, TEM images presented in Figure 4 show that the TiO_2_ nanoparticles in the Cu-AOL sample are larger than those observed in the Fe-AOL or AOL samples.

### 3.2. Chemical Analysis of Catalysts

The elemental composition of the catalysts was analyzed using X-ray fluorescence, as it was necessary to dehydrate and crush the fresh olive leaves before the analysis. As expected, carbon is the major element in the olive leaf (Table 1), representing 53.23 wt.%. In contrast, for the synthesized solids, where most of the carbon is removed from the samples, the majority component for the synthesized catalysts is Ti, with 53.2, 44.0 and 42.1%wt for AOL, Cu-AOL and Fe-AOL, respectively. This would mean that TiO_2_ accounts for 90, 83 and 74% of the weight of the synthesized catalysts, respectively. 

According to the synthetic procedure, if the Mg^2+^ exchange was complete (first stage), no Mg would remain in the synthesized catalysts. However, Table 1 shows that Mg percentage in the olive leaf is 0.1 wt.% and that there are no major differences with respect to the synthesized catalysts. However, the olive leaf presents 53.2% of the weight of C, which will almost be eliminated during the calcination process, therefore, the Mg content at this point will be higher. When considered that the total C is removed during the calcination process, the leaf presents 0.21 wt.% Mg, approximately twice the content of the uncalcined leaf. Consequently, half of the Mg content in the synthesized catalysts is reduced, but this element does not disappear completely, which may be due to the fact that the acid hydrolysis was not totally efficient, leaving part of the Mg in the catalysts. In the Cu-AOL and Fe-AOL catalysts the percentage of their respective dopant metal is significant and indicative of the presence of the metal in the catalyst, with a value of 2.78 wt.% Cu and 5.80 wt.% Fe. This shows that iron, as shown in the SEM micrographs, has a greater ability to enter the porphyrin rings, obtaining almost twice the content of the weight of copper.

It is interesting to note some of the essential elements in the olive leaves that were not removed in the hydrolysis process and that have been found in the synthesized catalysts. Calcium has remained in AOL with 1.83 wt.%, in Cu-AOL with 1.04 wt.% and in Fe-AOL with 0.88 wt.%; potassium is still present with 0.95, 0.84 and 0.72 wt.% for AOL, Cu-AOL and Fe-AOL catalysts, respectively; or phosphorus with 1.09 wt.% in AOL. The rest of the metals identified would be considered trace metals because of the small percentage quantified in the catalysts.

### 3.3. Structural Characterization of Catalyst

**Figure 5:**
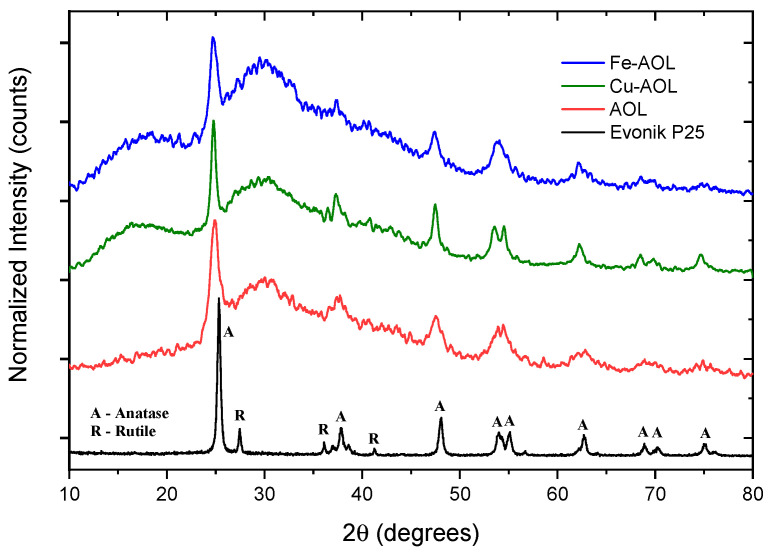
Diffractograms of a commercial titanium oxide, Evonik P25 and the synthesized solids, AOL, Fe-AOL, and Cu-AOL.

Figure 5 shows the diffractograms of the synthesized catalysts together with the diffractogram corresponding to the commercial Evonik P25, as a reference. Thus, P25 pattern shows narrow and well-defined peaks, related to a crystalline and ordered structure consisting of 80% anatase and 20% rutile crystalline phases. Therefore, diffraction peaks were identified at 25, 37, 48, 54, 55, 71 and 75° corresponding to the anatase phase crystallographic planes (101), (004), (200), (105), (211), (116), and (311), respectively [22,23]. In addition, diffraction peaks at 27, 36, and 41.2°, corresponding to the (110), (101), and (111) planes of rutile crystalline phase, could also be identified [24]. 

The diffractograms presented in Figure 5 show a series of broad bands with high background noise and with some narrower peaks appearing at 25, 37, 48 and 54° that are associated with the (101), (004), (200) and (105) planes of the anatase crystalline phase. The width of these peaks seems to indicate that TiO_2_ is in an incipient crystallization state, or that they are in the form of small anatase nanocrystals. Thus, the anatase peaks obtained for the Cu-AOL solid are narrower than those obtained for the AOL and Fe-AOL solids, which would indicate a higher degree of crystallinity or a larger crystallite size for this solid. The mean crystallite size data obtained from the Scherrer equation are 7.7, 6.7 and 6 nm for Cu-AOL, Fe-AOL and AOL, respectively, which is in agreement with the results obtained by TEM and discussed above (Figure 4). On the other hand, it was not possible to identify any characteristic peak of any crystalline copper or copper oxide phase in the Cu-AOL catalyst, nor any crystalline iron or iron oxide phase in the Fe-AOL catalyst.

**Figure 6:**
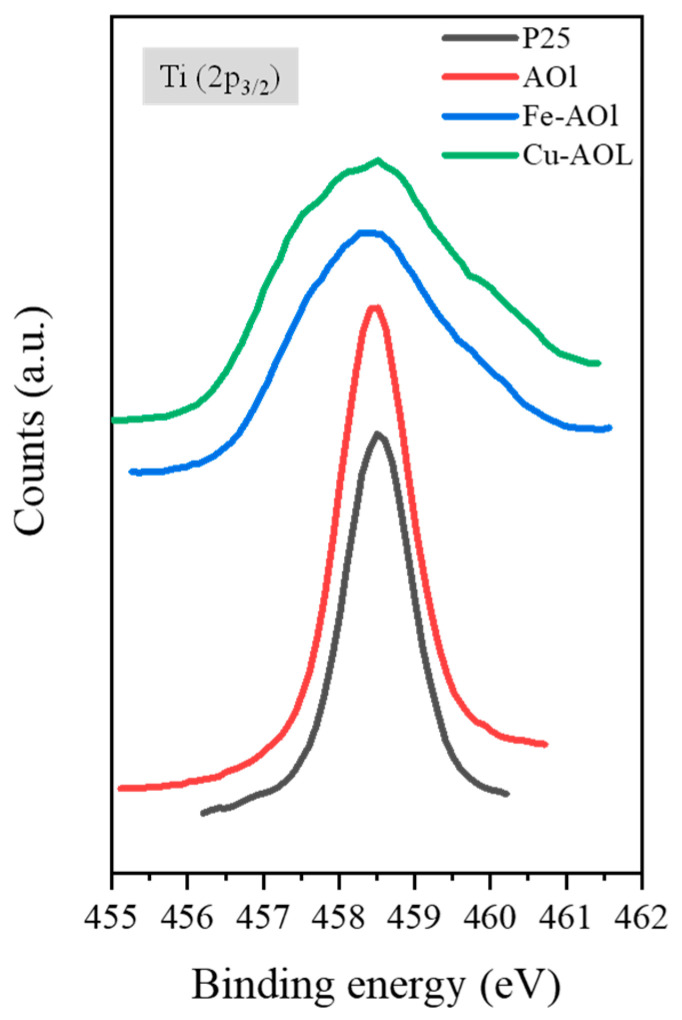
XPS spectrum of Ti (2p_3/2_) from a commercial titanium oxide, Evonik P25 and the synthesized solids, AOL, Fe-AOL, and Cu-AOL.

The surface chemical environment of the catalysts was analyzed using XPS. Figure 6 shows the XPS spectra of the commercial TiO_2_ Evonik P25 and the synthesized solids, where the spectra corresponding to the Fe-AOL and Cu-AOl solids are broader than those for Evonik P25 and AOL. Furthermore, in these catalysts, a peak shift towards lower binding energies is experienced, both with the incorporation of iron in Fe-AOL (−0.3 eV) and copper in Cu-AOL (−0.6 eV). As described by Cheng et al., this shift and band broadening to the half-peak is due to electron transfer from the metal (Fe or Cu) to TiO_2_ [25]. As can be seen, the titanium peak shift is more pronounced for copper, implying a higher electron transfer with TiO_2_ than iron. On the other hand, the different species were determined through deconvolution of the Ti 2p_3/2_ peaks (Figure S8). In Evonik P25 and AOL, two peaks were obtained after deconvolution, one at 457 eV binding energy corresponding to Ti^3+^ [26,27], and another at 458.5 eV associated with the Ti^4+^ species [26,27,28,29]. The Ti^3+^ species constitutes 4.28% of the total titanium obtained for Evonik P25, while in the AOL catalyst it accounts for 6.45%. Therefore, the synthesis, as proved in the study of Hidalgo et al., favors oxygen vacancies in the structure, generating defects [19]. In contrast to the AOL catalyst, only the peak corresponding to Ti^4+^ was identified in the Fe-AOL and Cu-AOL catalysts. Therefore, the Ti^3+^ found in the AOL catalyst could correspond to the titanium that has been introduced into the porphyrin group of chlorophyll.

**Figure 7:**
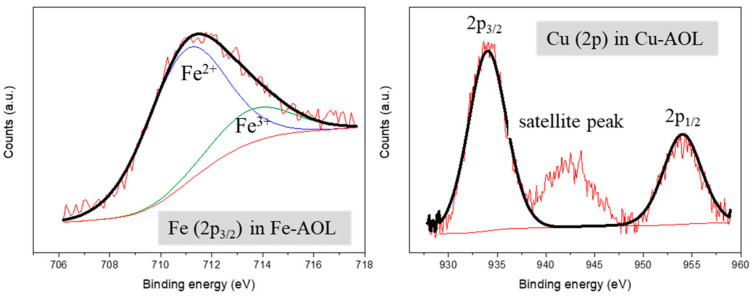
XPS spectra of Fe (2p_3/2_) and Cu (2p) for the Fe-AOL and Cu-AOL catalysts.

Figure 7 shows the XPS spectra of the Fe-AOL and Cu-AOL catalysts in the Cu and Fe regions. The Cu-AOL catalyst exhibits three peaks in the Cu region, one at a binding energy of 934 eV and another at 954 eV, corresponding to the Cu 2p_3/2_ and Cu 2p_1/2_ signals, respectively. The third peak is a satellite peak that has a binding energy of 942.3 eV and is characteristic of Cu^2+^ species [30], and therefore we can assume that copper is present in the catalyst as CuO. For this Cu-AOL solid, no Fe 2p_3/2_ signal was observed, although a small amount of iron was detected in XRF measurements. The copper amount determined in XPS is 2.81% by weight, a very similar value to that obtained using the XRF technique. 

As for the Fe-AOL spectrum, there is a signal at around 711.5 eV associated the Fe 2p_3/2_ that was deconvoluted in two peaks corresponding to Fe^2+^ (710.9 eV) and Fe^3+^ (713.5 eV) species, respectively [31,32]. Among both species, the most abundant was Fe^2+^ with 80%, while the overall iron content obtained using XPS accounted for 4.74%, a similar value to that reported using XRF. 

**Figure 8:**
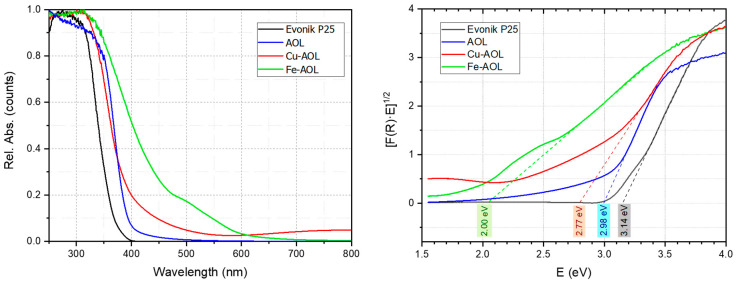
UV-Vis spectra AOL, Fe-AOL, Cu-AOL and Evonik P25 (**left**) and plot of the Kubelka-Munk function versus the energy of the absorbed light to determine the energy of the forbidden band (**right**).

To determine optoelectronic properties, the UV-Vis absorption spectra between 250 and 800 nm were obtained for the catalysts synthesized together with commercial Evonik P25 (Figure 8). For the Evonik P25, the UV-Vis spectra showed intense absorption below 400 nm due to the ligand-to-metal charge transfer (LMCT) associated with the O^2^⁻ to Ti^4+^ transitions. The band gap obtained for Evonik P25 from the Tauc’s plot (indirect transition model) was 3.14 eV (Figure 8). In the case of the AOL solid, there is a red shift of the absorption extending it to the 400–500 nm range. This extended absorption could be due to the higher amount of Ti^3+^ species or to the effect of the residual elements coming from the olive leaf (C, Ca, P, K, S, Si, Fe, among others) [33,34,35,36]. The observed red shift for AOL is reflected in a decrease in the band gap down to 2.98 eV, which could be associated with a higher percentage of Ti^3+^ species, which can generate a band that lies between the valence band and the conduction band that would extend the absorption of light into the visible range [29,37]. 

Furthermore, the incorporation of Fe and Cu in the synthesized solids further extends the absorption to the visible part of the spectrum, observing onsets of absorption at longer wavelength for Fe-AOL and Cu-AOL solids. Thus, in the case of Fe-AOL, there is a relevant red shift of the absorption in the 400–650 nm region that is usually ascribed in the literature to the d–d transitions of Fe^+3^ species [38]. The band gap for the Fe-AOL solid was 2.00 eV, a very intense decrease which is in agreement with the literature reports of high content iron-doped TiO_2_ catalysts [39]. Since, as observed in STEM-EDS micrographs, there is no iron oxides nanoparticles and data suggested that Fe is homogeneously distributed along the TiO_2_ structure, it could be assumed that some Fe^+3^ cations substituted the Ti^4+^ thus generating oxygen vacancies, thus expanding the light absorption to the visible light region and accounting for the observed band gap reduction. 

For the Cu-AOL solid, there was also a red-shift that was less intense in the 400–650 nm region but extended to the 650–800 nm region. There is some controversy in the bibliography about the origin of the extended absorption in the 400–550 nm region in Cu/TiO_2_ systems, being assigned to metallic Cu^0^, either through surface plasmon resonance (LSPR) or to d–s transitions Cu^0^ (d^10^s^1^), to the interfacial charge transfer (IFCT) from the TiO_2_ valence band (VB) to Cu^2+^ ions or to Cu^+^ metal-to-ligand charge transfer (MLCT) absorption [40]. In our case, we did not detect the presence of metallic nanoparticles in TEM measurements nor Cu^0^ in XPS measurements, so the effect of surface plasmon resonance or d–s transitions associated with metallic copper can be excluded. Moreover, the residual absorption observed above 550 nm and up to 800 nm in the Cu-AOL solid could be associated with d–d transitions in Cu^2+^ (d^9^s^0^). The band gap determined for this solid was 2.77 eV, much lower than that obtained for Evonik P25 and AOL solids. The narrowing of the band gap obtained from Tauc’s plot points to the presence of Cu inter-band gap states, suggesting that electronic communication between TiO_2_ and Cu was stablished.

The observed change in optical properties may be due to the formation of additional energy levels caused by impurities or dopant species that are located within the band gap of a semiconductor such as TiO_2_ NPs [41]. These localized defect states would produce an absorption tail that extends into the semiconductor’s bandgap. This absorption tail is known as the Urbach tail, and the associated energy is called the Urbach energy, E_U_ [42]. The Urbach energy, E_U_, is a parameter with energy dimensions that is used to quantify the energetic disorder at the edges of the energy bandgap of a semiconductor. It is, therefore, related to the steepness of the onset of absorption near the edge of the band, thus, related to the amplitude of the density of states and is useful to evaluate the energetic disorder of band edges in structurally disordered semiconductors. A sharper onset of absorption represents a lower Urbach energy. Urbach energy can be obtained by adjusting the absorption coefficient, α, as a function of energy within an exponential function (Equation (1)). Since absorption coefficient α is proportional to F(R), E_U_ can be obtained from the plot of Ln (FR) vs photon energy hν.

(1) $$\alpha \;={\;\alpha }_{0}\;\exp\left(\frac{h\nu }{E_{U}}\right)$$

The E_U_ values obtained from this plot (Supplementary Figure S9) are 74 meV for Evonik P25 and 304, 499 and 443 meV for AOL, Fe-AOL and Cu-AOL, respectively. These results indicate that the band gap boundary energy levels for Evonik P25 TiO_2_ are quite sharp, as compared to those of synthesized artificial olive leaves, AOL, Fe-AOL and Cu-AOL. This would be in agreement with an increase in energy states at the edges of such energy limits as a consequence of the presence of residual metals or, particularly, to the Fe and Cu ions incorporated during the synthesis process and which have presumably been incorporated into the TiO_2_ structure formed during the sol-gel and subsequent calcination processes. TEM, SEM and XPS proved the absence of metal or metal oxide nanoparticles but the incorporation of copper or iron cations on the TiO_2_ structure, thus leading to the red shift of the absorption and changes in Eg and E_U_ values.

### 3.4. Photo-Catalytic Experiments

**Figure 9:**
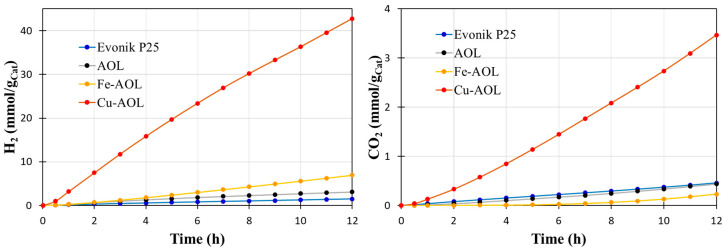
shows the accumulated production of hydrogen from the photo-reforming of aqueous glycerol solutions (10% *v/v*) under UV irradiation with the catalysts synthesized in this study, including the commercial solid Evonik P25 as a reference.

To probe the photocatalytic efficiency of the synthesized catalysts by replicating the olive leaf structure with TiO_2_, Fe-TiO_2_ and Cu-TiO_2_, the glycerol photo-reforming reaction was carried out under solar and UV light. Figure 9 shows the data obtained in the hydrogen and carbon dioxide photoproduction for Evonik P25 and the synthesized catalysts under UV light. The results indicate that all the solids synthesized improve to P25, although in an uneven way. Thus, the solid AOL made up solely of TiO_2_ leads to a slight improvement compared to P25, with an accumulated production of hydrogen after 12 h of reaction of 3.12 mmolH_2_/g_Cat_ compared to the 1.45 mmol H_2_/g_cat_ produced with commercial TiO_2_. On the other hand, the incorporation of Fe improves the hydrogen production capacity by producing 6.94 mmol H_2_/g_Cat_, which implies doubling the hydrogen production compared to AOL. However, the most relevant results are those obtained by the Cu-AOL catalyst that produces 42.8 mmolH_2_/g_Cat_ after 12 h of reaction, which represents a hydrogen production almost 30 times higher than that of P25 and 14 times more than AOL.

**Figure 10:**
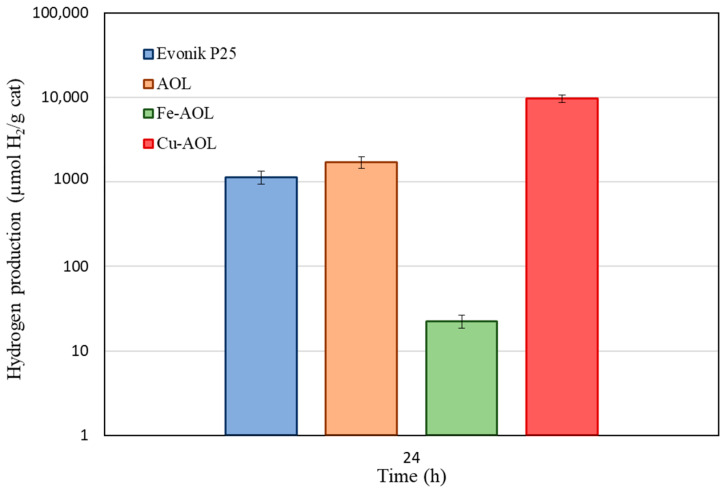
Hydrogen photoproduction by Evonik P25, AOL, Fe-AOL and Cu-AOL under sunlight during 24 h.

Figure 10 summarizes the total hydrogen production of the commercial TiO_2_ and the synthesized catalysts after being under solar irradiation for 24 h. Contrary to what occurs under UV radiation, when illuminated by a solar simulator, the catalysts do not exhibit the same behavior. As can be seen in this figure, the AOL catalyst also improves hydrogen production with respect to the commercial Evonik P25, with a production of 1.7 mmol H_2_/g_Cat_ versus 1.1 mmolH_2_/g_Cat_ for P25. The Fe-AOL catalyst, on the contrary, when working under solar radiation, is the catalyst with the lowest activity (22.5 µmol H_2_/g_Cat_), being even less active than the reference Evonik P25. The catalyst with the highest catalytic activity continues to be Cu-AOL with a production of 9.7 mmol H_2_/g_Cat_, 9 times higher than Evonik P25 and 6 times higher than the AOL catalyst.

The improved activity of the AOL catalyst with respect to P25 may be due, as seen in XPS, to the high percentage of Ti^3+^ species, which implies a higher content of defects in the structure that can slow down the recombination rate of the e^−^/h^+^ pairs [19]. Moreover, as was verified by UV-visible spectroscopy, the band gap of this catalyst is smaller, displacing the light absorption towards the visible region and having a higher energetic disorder at the band edges than Evonik P25. The Fe-AOL catalyst best mimics the leaf structure, therefore higher activity would be expected since it could enhance light diffusion through the replicated structures of the olive leaf. However, although under UV radiation it increased the activity as compared to AOL, under solar radiation it presented the lowest activity among the synthesized catalysts. This could be because Fe has a high exchange facility in the porphyrin group of chlorophyll due to its similarity with the porphyrin group of hemoglobin, so it reaches a high amount of this metal in the final catalyst. However, this is disadvantageous from the catalytic point of view, since it has been proved that iron, at high concentrations, can act as a recombination center of the photogenerated electrons and holes, due to the decrease of the distance between the capture sites, thus causing a decrease in its activity [43,44,45,46,47]. 

Finally, Cu-AOL solid is the best-performing photocatalyst among the synthesized ones, both under UV and solar radiation. As confirmed using the XPS technique, the Cu incorporated during the synthesis is not in the metallic state but forms copper (II) oxide. Therefore, we would be facing a p-n heterojunction between two semiconductors, where the charge carriers can separate rapidly, causing a decrease in the recombination rate of the e^-^/h^+^ pairs of TiO_2_, thus improving the photocatalytic activity. Transferring electrons from the conduction band of TiO_2_ to the conduction band of CuO, where the H^+^ to H_2_ reduction process would take place. This electron transfer between CuO and TiO_2_ could be the cause of the Ti 2p_3/2_ band broadening and band shift in XPS. Comparing the hydrogen production of this catalyst with its homonym synthesized only based on Ti (AOL), it presents a 14-fold higher production when working with UV radiation and 6-fold higher when working under solar radiation. This improvement in hydrogen production under solar radiation is due to the fact that the heterojunction formed in the Cu-AOL catalyst shifts the absorption spectrum of the catalyst towards the visible light range, and it has also been demonstrated in numerous studies that CuO is a very good co-catalyst of TiO_2_ [20,48,49,50,51].

In order to compare the photocatalytic activity of the synthesized catalysts with those found in the literature, it is necessary to standardize the results. Since each author works with a different experimental procedure, sacrificial agent, conditions and light sources. For this purpose, the increase in the activity of each catalyst with respect to TiO_2_ (as reference) is calculated. In this work the activity of Cu-AOL and Fe-AOL is 30 times and 5 times higher than TiO_2_ under UV radiation. When compared with some similar catalysts found in the literature (Table 2), very promising results are obtained with these new catalysts. In a previous study with physical mixtures of CuO and TiO_2_ under UV light, 5% CuO could only enhance the activity of TiO_2_ 8-fold, compared to 30-fold for the Cu-AOL catalyst [49]. Hinojosa-Reyes et al. synthesized a composite with 1% copper but were only able to increase the TiO_2_ activity 5-fold under UV light [52]. In the case of iron and titanium-based catalysts, Valášková et al. synthesized a composite that was able to improve TiO_2_ only 1.3 times [53], while Bootluck et al. synthesized an iron-based catalyst that increased the activity of TiO_2_ 18 times under UV light [54]. Fe-AOL catalytic behavior is between these two catalysts, improving H_2_ production with respect to TiO_2_ but not very significantly. Under sunlight, however, the activity of the Fe-AOL catalyst is very low, while that of Cu-AOL increases 9 times that of TiO_2_. In the literature, on the other hand, catalysts based on copper and titanium increase the activity with respect to TiO_2_ approximately 3 times with different sacrificial agents, glycerol [55], ethanol [56] and methanol [57].

The improvement of photocatalytic activity in hydrogen production from glycerol reforming with these new photocatalysts is not the only applicability of the catalysts. They could be used in CO_2_ reduction [58,59], decontamination of water from oxygenated organic compounds [59,60], dyes [61,62,63,64], among other photocatalytic applications [63,65,66].

## 4. Conclusions

Fe is the metal that allows the most accurate replication of all leaf channels due to its ability to be incorporated into the porphyrin group of the chlorophyll. The XPS technique allowed us to demonstrate that the AOL catalyst presents both Ti^3+^ and Ti^4+^ species, probably due to the exchange of Ti^3+^ in the porphyrin ring, while in its Fe and Cu counterparts only Ti^4+^ is identified, although with a broadening of the peak and a displacement of the binding energy due to the electronic transfer between the metal and titanium, more pronounced in the case of copper, which implies a greater electronic transfer. With this technique, it was also possible to verify that the Cu-AOL catalyst presents copper as Cu^2+^, while in the Fe-AOL catalyst there is a mixture between Fe^2+^ and Fe^3+^, with Fe^2+^ being the main species. The UV-Vis absorption spectra showed how the catalysts present an absorption spectrum shifted towards the visible region, much more marked in catalysts containing copper and iron as co-catalysts, which implies a decrease in the bandgap of the semiconductor and is associated with a higher photocatalytic performance.

The catalysts were tested in the glycerol photo-reforming reaction to produce hydrogen under both UV and sunlight. The use of iron, despite being the metal that best enables leaf replication, achieves improved activity under UV light, but not under sunlight, probably due to an excess of Fe in the structure. By contrast, Cu is shown to be a great co-catalyst of TiO_2_ and achieves the highest activity of the synthesized catalysts (42.8 and 9.7 mmol H_2_/g_Cat_ under UV and solar light, respectively).

## Figures and Tables

**Table 1:** Elemental composition obtained using XRF of the synthesized catalysts and olive leaf.

Elements	Olive Leaf (% Weight)	AOL (% Weight)	Cu-AOL (% Weight)	Fe-AOL (% Weight)
Ti	0	53.20	44.00	42.13
Fe	0.40	0.05	0.04	5.80
Cu	0	0	2.78	0
Mg	0.10	0.12	0.07	0.08
Al	0.03	0.08	0.03	0.06
Si	0.06	0.38	0.10	0.53
P	0.16	1.09	0.11	0.22
S	0.17	0.73	0.61	0.76
Cl	0.11	0.01	0.48	0.27
K	1.27	0.95	0.84	0.72
Ca	1.59	1.83	1.04	0.88
C	53.23	2.28	1.99	2.81

**Table 2:** Comparison of hydrogen photoproduction with self and reported catalysts.

Catalyst	Sacrificial Agent	Light Source	Hydrogen Production (TiO_2_) (mmol/h·g_Cat_)	Hydrogen Production (Catalyst) (mmol/h·g_Cat_)	Reference
Cu-AOL	Glycerol at 10%	UV	1.4	42.8	This study
5% CuO-TiO_2_	Glycerol at 10%	UV	0.4	3.24	[49]
1% Cu-TiO_2_	Ethanol at 50%	UV	0.96	3.86	[52]
Fe-AOL	Glycerol at 10%	UV	1.4	6.94	This study
Fe_2_O_3_-TiO_2_	Methanol at 30%	UV	0.04	0.74	[54]
Fe_2_O_3_-TiO_2_	Methanol at 50%	UV	1	1.3	[53]
Cu-AOL	Glycerol at 10%	Solar	1.1	9.7	This study
5% CuO-TiO_2_	Glycerol at 2.8%	Visible	0.2	0.6	[55]
CuO-TiO_2_	Ethanol at 10%	Solar	1.9	7	[56]
CuO-TiO_2_	Methanol at 20%	Solar	3.4	7.2	[57]

## Data Availability

Data will be available on request.

## Supplementary Materials

The following supporting information can be downloaded at: https://www.mdpi.com/article/10.3390/nano13040664/s1. Additional figures are supplied as supplementary materials: Figure S1: (**A**) secondary electron detector (SEM), (**B**) backscattered electron (BSE) and (**C**) Ti and O energy-dispersive X-ray spectroscopy (EDX) micrographs of AOL; Figure S2: (**A**) secondary electron detector (SEM), (**B**) backscattered electron (BSE) and (**C**) Ti and Cu energy-dispersive X-ray spectroscopy (EDX) micrographs of Cu-AOL; Figure S3: (**A**) secondary electron detector (SEM), (**B**) backscattered electron (BSE) and (**C**) Ti and Fe energy-dispersive X-ray spectroscopy (EDX) micrographs of Fe-AOL; Figure S4: (**A**) transmission electron microscopy (TEM), (**B**) scanning transmission electron microscopy (STEM) and (**C**) Cu and Ti energy-dispersive X-ray spectroscopy (EDX) micrographs of Cu-AOL; Figure S5: (**A**) transmission electron microscopy (TEM), (**B**) scanning transmission electron microscopy (STEM) and (**C**) Cu and Ti energy-dispersive X-ray spectroscopy (EDX) micrographs of Cu-AOL; Figure S6: (**A**) transmission electron microscopy (TEM), (**B**) scanning transmission electron microscopy (STEM) and (**C**) Cu and Ti energy-dispersive X-ray spectroscopy (EDX) micrographs of Fe-AOL; Figure S7: (**A**) transmission electron microscopy (TEM), (**B**) scanning transmission electron microscopy (STEM) and (**C**) Cu and Ti energy-dispersive X-ray spectroscopy (EDX) micrographs of Fe-AOL; Figure S8: XPS spectrum and deconvolution of Ti (2p3/2) of (**a**) Evonik P25, (**b**) AOL, (**c**) Fe-AOL and (**d**) Cu-AOL; Figure S9: Urbach Energy (E_U_) obtained for the catalysts synthesized in this study. Evonik P25 was included as reference. The Urbach energy is obtained from the inverse of the slope of the Ln F(R) vs Energy plot.
