# Supplementary material for: Synthesis of Fe-TiO2 and Cu-TiO2 Based Materials by Olive Leaves Biotemplating—Application to Hydrogen Production from Glycerol Photoreforming
Source: Nanomaterials (Basel). 2023 Feb 8;13(4):664. doi: 10.3390/nano13040664 (PMC9966289; doi:10.3390/nano13040664)
Supplement: Supplementary file 1 [file nanomaterials-13-00664-s001.zip › nanomaterials-2195340-supplementary.pdf]

Supporting information

Fe-TiO_2 and Cu-TiO_2 based materials from olive leaves biotemplating. Synthesis and application to hydrogen production from glycerol photoreforming.

Juan Martín-Gómez[a,*], Susana Reca-Expósito[a], Francisco J. López-Tenllado, Jesús Hidalgo-Carrillo[a], Alberto Marinas[a], Francisco J. Urbano[a]

[a] Departamento de Química Orgánica, Instituto Químico para la Energía y el Medioambiente (IQUEMA), Universidad de Córdoba, E-14071 Córdoba, Spain

*Corresponding author: Juan Martín-Gómez (q92magoj@uco.es)

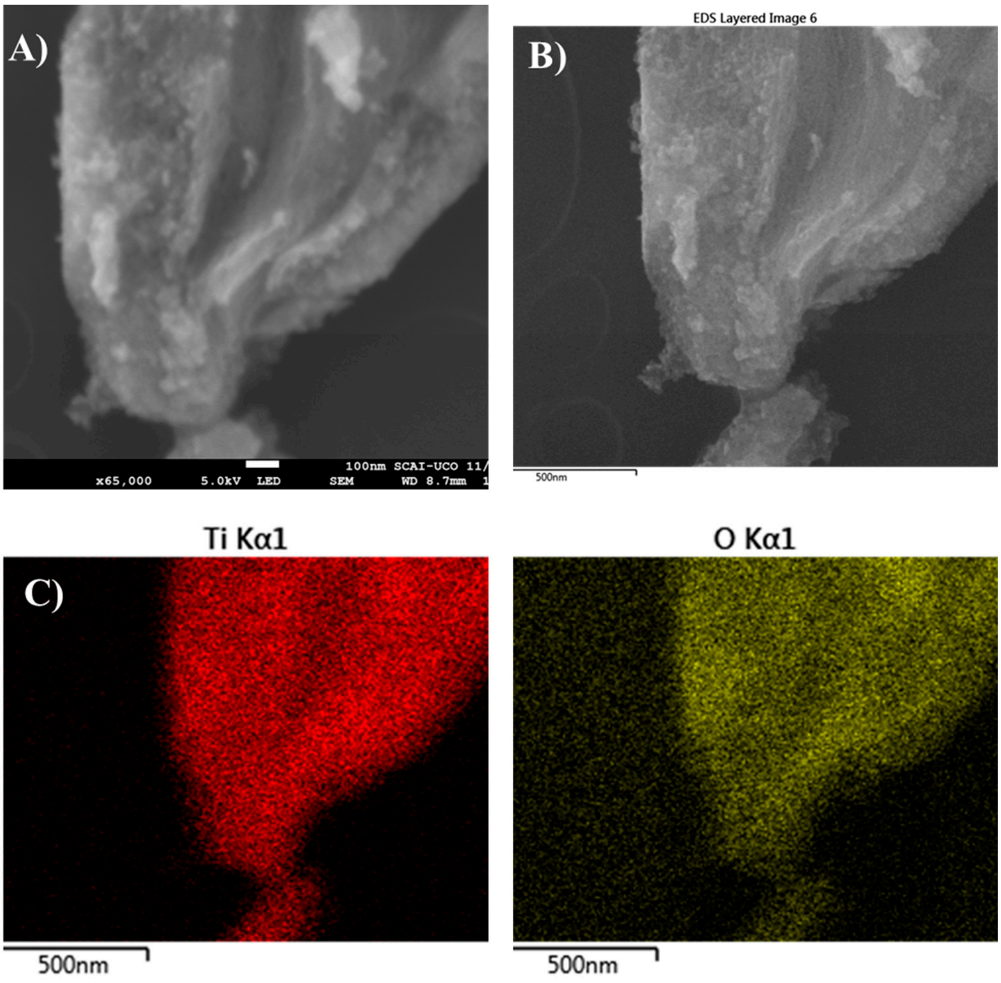

Figure S1. (A) Secondary electron detector (SEM), (B) backscattered electron (BSE) and (C) Ti and O energy-dispersive X-ray spectroscopy (EDX) micrographs of AOL.

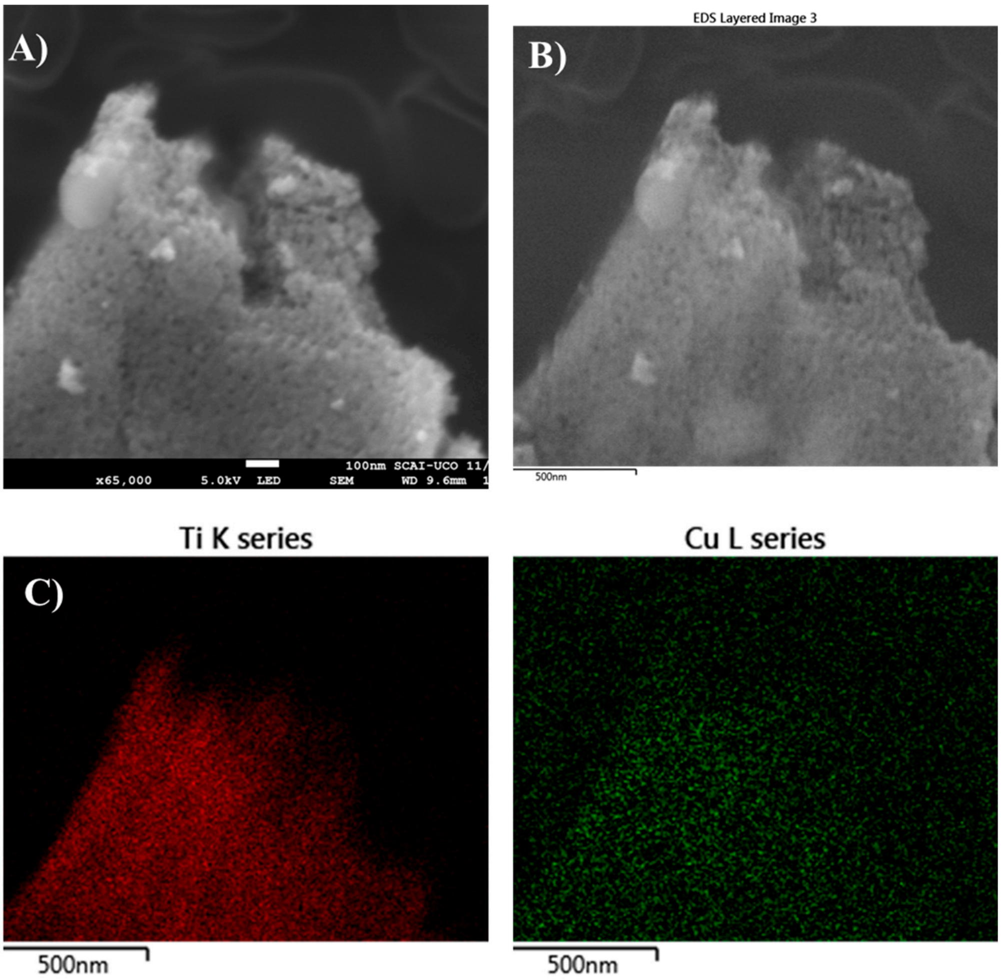

Figure S2. (A) Secondary electron detector (SEM), (B) backscattered electron (BSE) and (C) Ti and Cu energy-dispersive X-ray spectroscopy (EDX) micrographs of Cu-AOL.

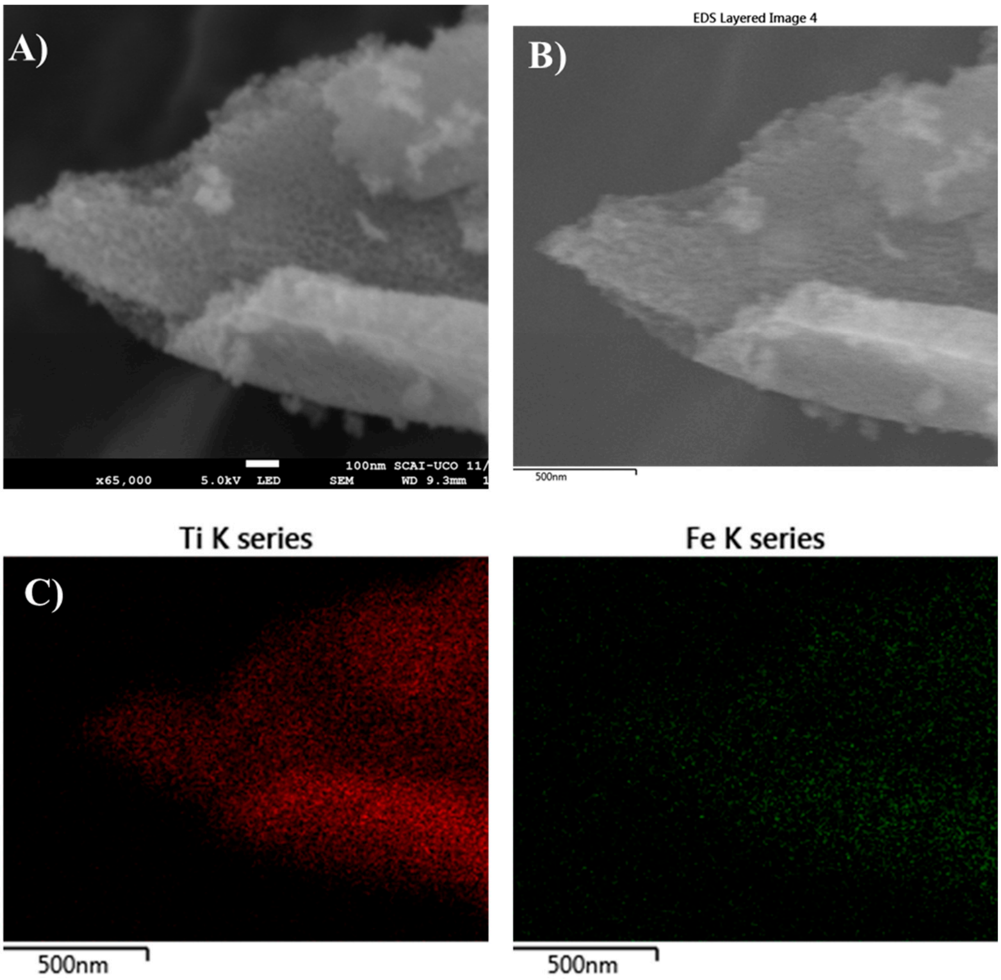

Figure S3. (A) Secondary electron detector (SEM), (B) backscattered electron (BSE) and (C) Ti and Fe energy-dispersive X-ray spectroscopy (EDX) micrographs of Fe-AOL.

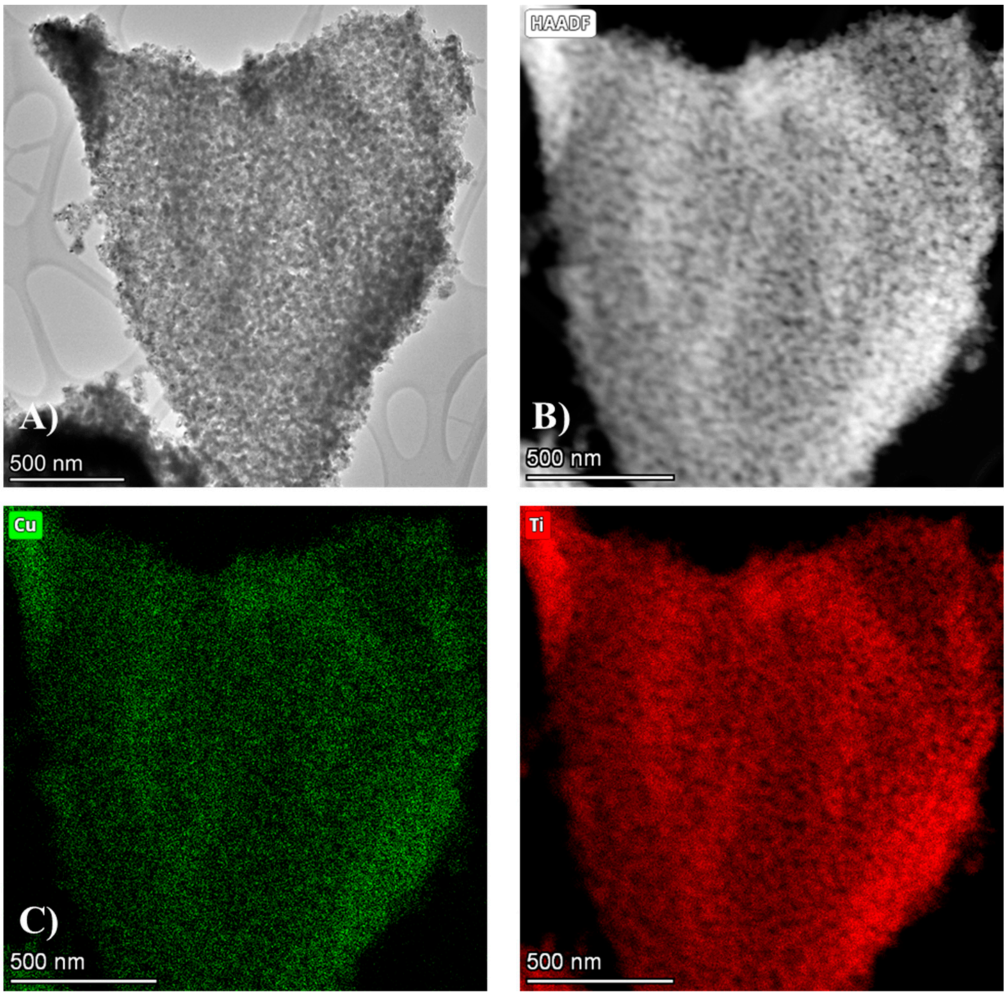

Figure S4. (A) Transmission Electron Microscopy (TEM), (B) Scanning Transmission Electron Microscopy (STEM) and (C) Cu and Ti energy-dispersive X-ray spectroscopy (EDX) micrographs of Cu-AOL.

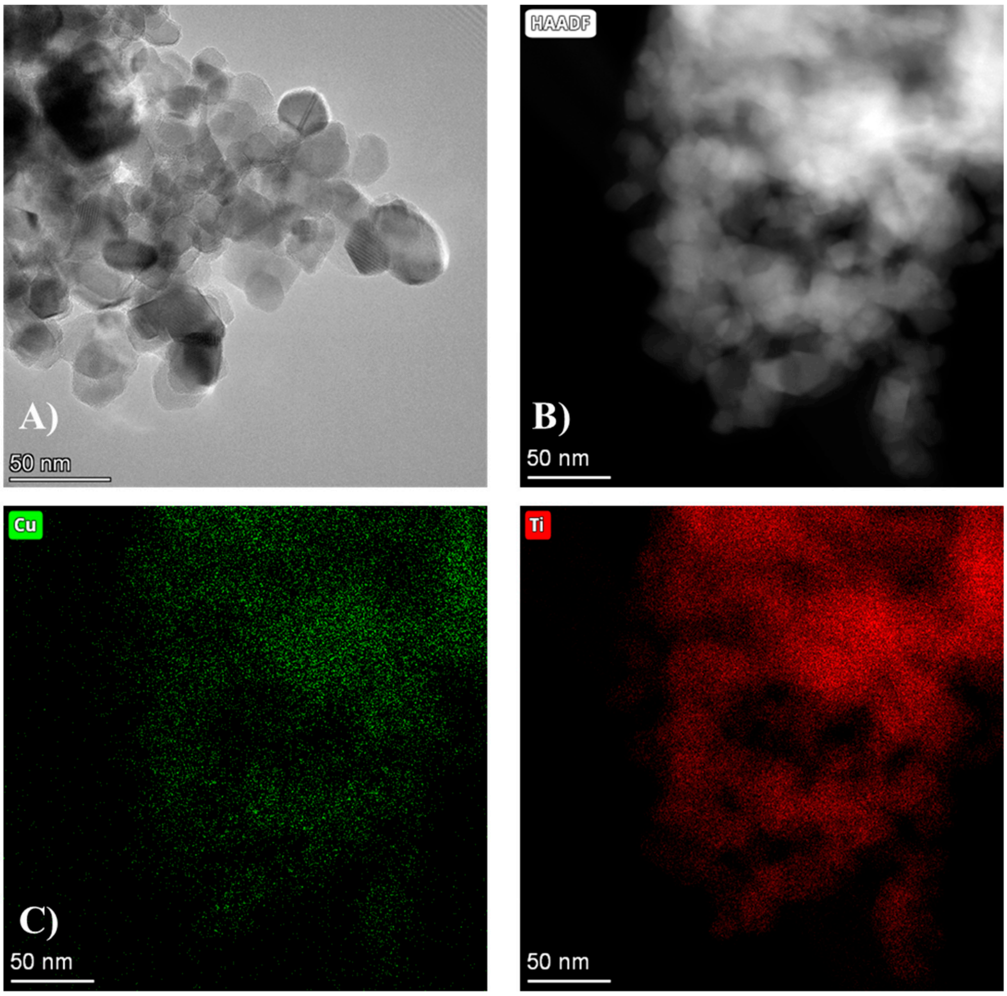

Figure S5. (A) Transmission Electron Microscopy (TEM), (B) Scanning Transmission Electron Microscopy (STEM) and (C) Cu and Ti energy-dispersive X-ray spectroscopy (EDX) micrographs of Cu-AOL.

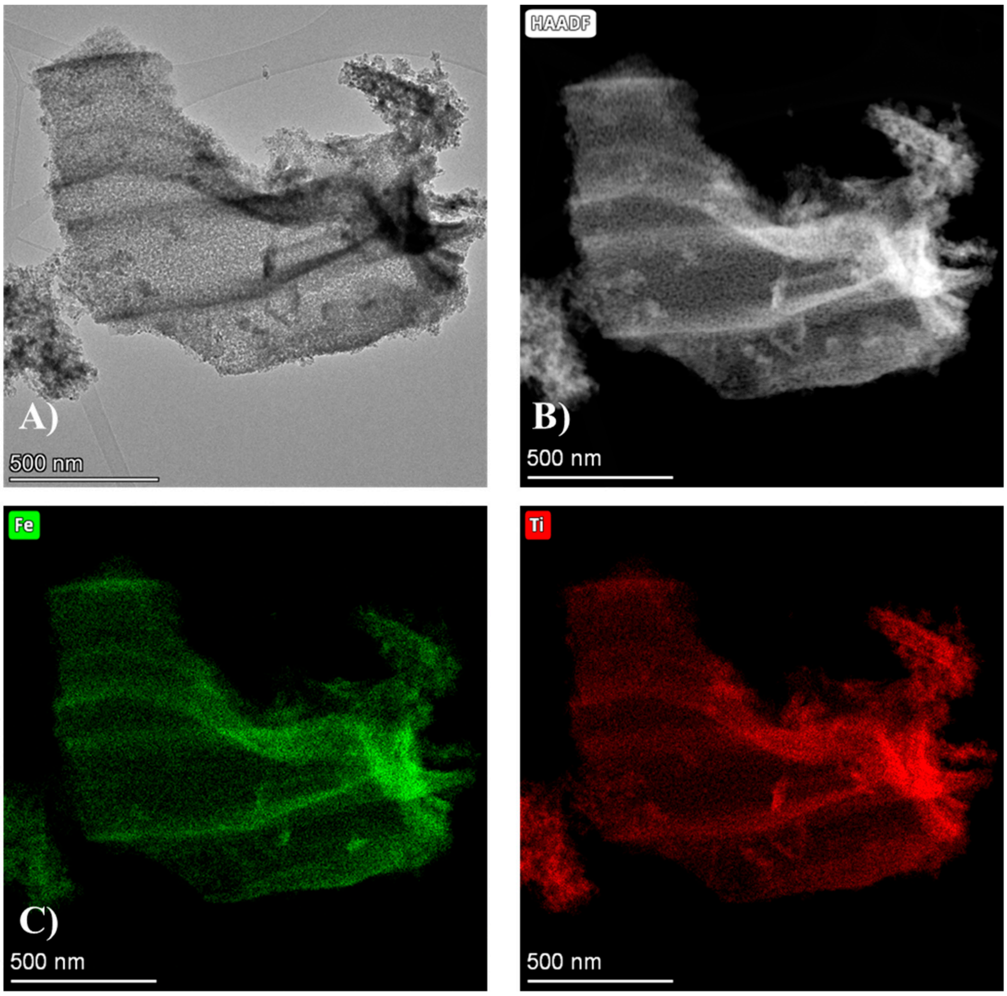

Figure S6. (A) Transmission Electron Microscopy (TEM), (B) Scanning Transmission Electron Microscopy (STEM) and (C) Cu and Ti energy-dispersive X-ray spectroscopy (EDX) micrographs of Fe-AOL.

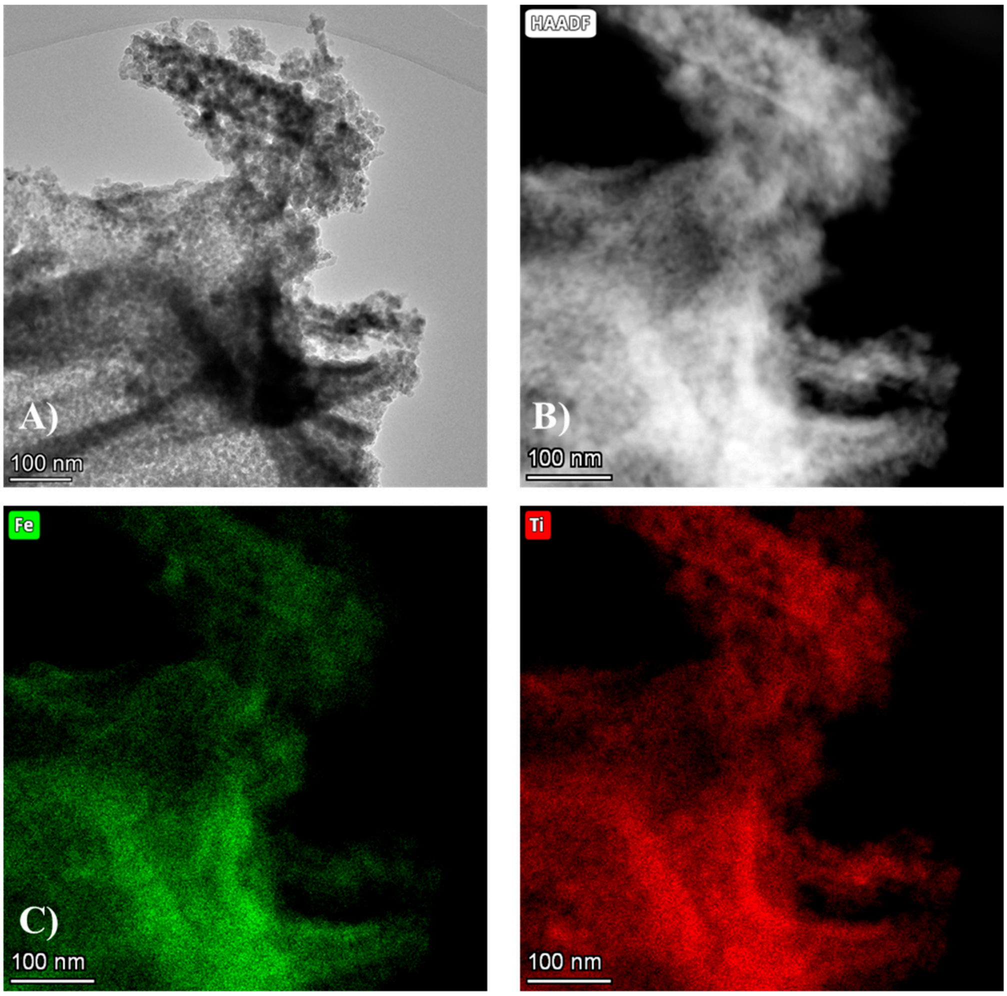

Figure S7. (A) Transmission Electron Microscopy (TEM), (B) Scanning Transmission Electron Microscopy (STEM) and (C) Cu and Ti energy-dispersive X-ray spectroscopy (EDX) micrographs of Fe-AOL.

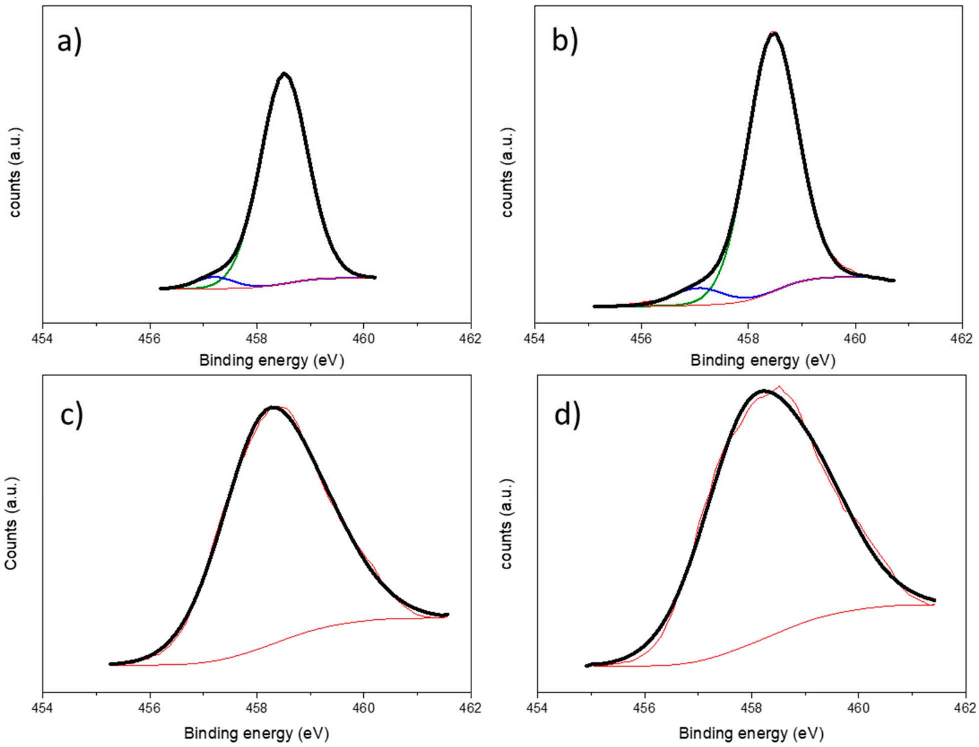

Figure S8. XPS spectrum and deconvolution of Ti ($2p_{3/2}$) of (a) Evonik P25, (b) AOL, (c) Fe-AOL and (d) Cu-AOL.

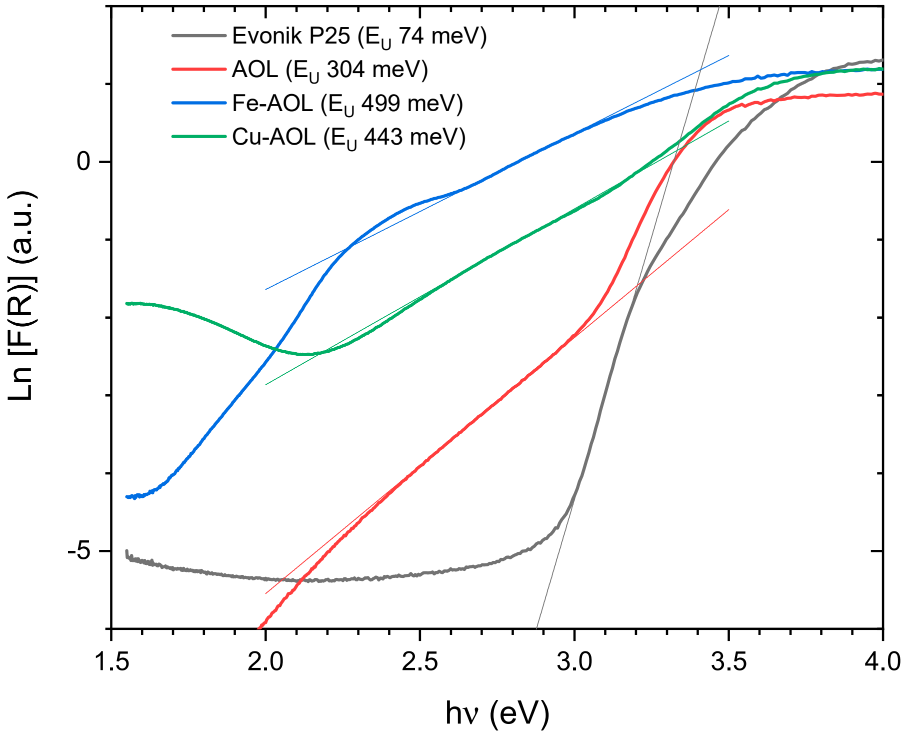

Figure S9. Urbach Energy (EU) obtained for the catalysts synthesized in this work. Evonik P25 was included as reference. The Urbach energy is obtained from the inverse of the slope of the Ln F(R) vs Energy plot.
